# Detection of adamantane-sensitive influenza A(H3N2) viruses in Australia, 2017: a cause for hope?

**DOI:** 10.2807/1560-7917.ES.2017.22.47.17-00731

**Published:** 2017-11-23

**Authors:** Aeron Hurt, Naomi Komadina, Yi-Mo Deng, Matthew Kaye, Sheena Sullivan, Kanta Subbarao, Ian Barr

**Affiliations:** 1 WHO Collaborating Centre for Reference and Research on Influenza, The Peter Doherty Institute for Infection and Immunity, Melbourne, Victoria, Australia; 2 Department of Microbiology and Immunology, The University of Melbourne, The Peter Doherty Institute for Infection and Immunity, Melbourne, Victoria, Australia; 3 School of Global and Population Health, The University of Melbourne, Victoria, Australia

**Keywords:** influenza virus, adamantane resistance, amantadine, antiviral

## Abstract

For over a decade virtually all A(H3N2) influenza viruses have been resistant to the adamantane class of antivirals. However, during the 2017 influenza season in Australia, 15/461 (3.3%) adamantane-sensitive A(H3N2) viruses encoding serine at residue 31 of the M2 protein were detected, more than the total number identified globally during the last 6 years. A return to wide circulation of adamantane-sensitive A(H3N2) viruses would revive the option of using these drugs for treatment and prophylaxis.

Amantadine and rimantadine are compounds of the adamantane class of antivirals which act on influenza A viruses by binding to the M2 ion channel, preventing uncoating of the virus during replication. Treatment of influenza A virus infection with these drugs within 48 hours of symptom onset reduces illness by ca 24 hours, and when given prophylactically, the drugs can prevent ca 60% of influenza cases [1]. However, effectiveness of both drugs is lost when viruses acquire an amino acid substitution at one of five critical residues of the M2 protein i.e. positions 26, 27, 30, 31 and 34 [2]. The occurrence of adamantane-resistant influenza A viruses was rare among circulating influenza viruses [3] until 2000, when an increasing proportion of viruses from Asia, particularly China, contained the serine (S) to asparagine (N) substitution at residue 31 (S31N) of the M2 protein [4]. By the end of the 2005/06 influenza season, over 90% of circulating A(H3N2) viruses in North America and other parts of the northern hemisphere, such as Asia, contained the S31N substitution even though the vast majority of resistant viruses were from patients who had not been treated with adamantanes [5]. After more than 7 years of almost complete dominance of adamantane-resistant A(H3N2) influenza viruses globally, we describe the detection in Australia of increased numbers of adamantane-sensitive viruses during the 2017 influenza season.

## Global frequency of adamantane-resistant A(H3N2) viruses 1968–2017

Analysis of all M2 gene sequences of A(H3N2) viruses deposited in the EpiFlu public sequence database of the Global Initiative on Sharing All Influenza Data (GISAID) (n = 26,231; as at 21 November 2017), showed that the global frequency of adamantane-resistant A(H3N2) viruses with M2 N31 has been > 99% each year from 2010 to 2017 (Table 1).

**Table 1:** Frequency of influenza A(H3N2) viruses with different amino acids at residue 31 of the M2 protein, 1968–2017^a^

Year(s)	M2 S31 (adamantane-sensitive)		M2 N31 (adamantane-resistant)		M2 D31 (adamantane-resistant)	
	n	%	n	%	n	%
1968–1999	1,153	98.3	20	1.7	0	0.0
2000	224	99.1	2	0.9	0	0.0
2001	112	99.1	1	0.9	0	0.0
2002	304	99.0	3	1.0	0	0.0
2003	424	87.8	59	12.2	0	0.0
2004^b^	341	77.5	98	22.3	0	0.0
2005	229	45.1	279	54.9	0	0.0
2006	38	13.0	254	87.0	0	0.0
2007	85	12.6	589	87.0	3	0.4
2008	7	1.2	597	98.8	0	0.0
2009	11	1.1	948	98.9	0	0.0
2010	9	0.9	973	99.1	0	0.0
2011	3	0.2	1,307	99.7	1	0.1
2012	3	0.2	1,897	99.8	0	0.0
2013	0	0.0	1,445	100.0	0	0.0
2014	2	0.1	1,892	99.6	5	0.3
2015	2	0.1	3,454	99.9	1	< 0.1
2016	1	< 0.1	4,286	> 99.9	1	< 0.1
2017^c^	20	0.4	5,142	99.5	5	0.1

^a^ As at 21 November 2017.

^b^ One virus with M2 I31 was detected.

^C^ Includes the Australian viruses reported in this study.

Percentages rounded to one decimal.

The authors acknowledge the originating and submitting laboratories that provided sequences to GISAID’s EpiFlu database which were used to calculate the global frequencies of A(H3N2) viruses with different amino acid residues at position 31 of the M2 (www.gisaid.org).

## Analysis of the adamantane-resistance situation in Australia during the 2017 influenza season

The Australian 2017 influenza season was dominated by high levels of A(H3N2) influenza virus activity. During this season, 15 adamantane-sensitive A(H3N2) viruses encoding M2 S31 were detected in Australia (Table 2), which exceeded the cumulative total of 11 adamantane-sensitive influenza A viruses detected globally between 2011 and 2016 (Table 1). In contrast, the frequency of adamantane-resistance in circulating A(H1N1)pdm09 viruses has remained unchanged at > 99.9% both in Australia and worldwide.

**Table 2:** Details of M2 S31 and D31 influenza A(H3N2) viruses detected in Australia and New Zealand, July –September 2017 (n=21)

Virus designation	Location	Specimen date (2017)	M2 31 residue	Age (years)	Sex	Patient status	GISAID isolate ID
A/Wellington/35/2017	Wellington, New Zealand	3 Jul	S	Unknown	Female	Out-patient	EPI_ISL_277612
A/Victoria/27/2017	Victoria, Australia	3 Jul	D	20	Female	Unknown	EPI_ISL_277591
A/Victoria/22/2017	Victoria, Australia	4 Jul	S	21	Female	Unknown	EPI_ISL_275249
A/Victoria/1008/2017	Victoria, Australia	18 Jul	S	27	Male	Unknown	EPI_ISL_277586
A/Victoria/51/2017	Victoria, Australia	18 Jul	D	77	Male	Unknown	EPI_ISL_277338
A/Victoria/586/2017	Victoria, Australia	21 Jul	S	Unknown	Unknown	Unknown	EPI_ISL_278020
A/Victoria/628/2017	Victoria, Australia	4 Aug	S	90	Male	Inpatient	EPI_ISL_277968
A/Victoria/2069/2017	Victoria, Australia	4 Aug	D	57	Male	Unknown	EPI_ISL_277965
A/Brisbane/1017/2017	Queensland, Australia	7 Aug	S	31	Female	Outpatient	EPI_ISL_277915
A/Sydney/1101/2017	New South Wales, Australia	9 Aug	S	84	Female	Unknown	EPI_ISL_283125
A/Sydney/1079/2017	New South Wales, Australia	9 Aug	S	30	Male	Unknown	EPI_ISL_277907
A/Victoria/653/2017	Victoria, Australia	10 Aug	S	23	Female	Outpatient	EPI_ISL_277940
A/Victoria/655/2017	Victoria, Australia	10 Aug	S	82	Male	Outpatient	EPI_ISL_277969
A/Victoria/624/2017	Victoria, Australia	11 Aug	S	82	Male	Inpatient	EPI_ISL_277922
A/Brisbane/140/2017	Queensland, Australia	22 Aug	S	2 months	Female	Outpatient	EPI_ISL_283124
A/Darwin/1016/2017	Northern Territory, Australia	31 Aug	S	42	Male	Unknown	EPI_ISL_283121
A/Victoria/708/2017	Victoria, Australia	2 Sep	D	8	Female	Outpatient	EPI_ISL_283122
A/Victoria/697/2017	Victoria, Australia	2 Sep	S	77	Male	Inpatient	EPI_ISL_283119
A/Victoria/856/2017	Victoria, Australia	18 Sep	D	9	Female	Unknown	Poor sequence quality
A/Victoria/211/2017	Victoria, Australia	19 Sep	S	68	Male	Deceased	EPI_ISL_283123
A/Victoria/872/2017	Victoria, Australia	26 Sep	S	58	Female	Unknown	EPI_ISL_283120

**Figure 1:**
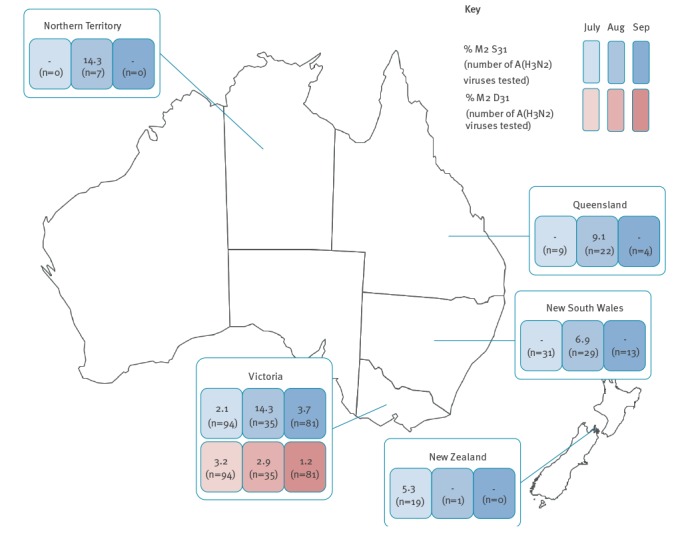
Frequency of M2 S31 and D31 viruses among influenza A(H3N2) viruses, Australia and New Zealand, July–September 2017

Using Sanger sequencing [6], next generation sequencing [7] or pyrosequencing [8] we found that from 1 March to 30 June 2017, all 310 tested Australian A(H3N2) viruses encoded M2 N31 indicating adamantane-resistance (M2 N31 viruses). However, in July, August and September 2017, two of 201 (1.0%), 10 of 115 (8.7%) and three of 145 (2.1%) tested A(H3N2) virus isolates, respectively, encoded M2 S31 indicating adamantane-sensitivity (M2 S31 viruses). The M2 S31 viruses were detected across four states in Australia (Victoria: n = 10; New South Wales: n = 2; Queensland: n = 2; Northern Territory: n = 1), as well as 1 of 70 tested from New Zealand (Figure 1), indicating that although overall frequency was low, the M2 S31 viruses had spread across a large geographic area (Table 2). A(H3N2) influenza viruses tested from the other Australian states of South Australia (n = 148), Western Australia (n = 40), Tasmania (n = 54) and the Australian Capital Territory (n = 32) were all M2 N31 viruses.

No M2 S31 or M2 D31 viruses were detected in the states of South Australia, Western Australia, Tasmania or the Australian Capital Territory where the number of influenza A (H3N2) viruses tested was 149, 40, 52 and 32, respectively.

In Victoria, M2 S31 viruses were detected in July, August and September, with the peak detection frequency of five of 35 occurring in August (Figure 1). In addition to the 10 M2 S31 viruses, a further five Victorian A(H3N2) viruses contained an alternative amino acid, aspartic acid (D), at residue 31 of the M2 protein (M2 D31 viruses) (Figure 1)(Table 2). M2 D31 viruses have been extremely rare since the emergence of the A(H3N2) viruses in 1968, with just 11 of 21,064 (0.05%) viruses detected globally before 2017, compared with the detection rate of five of 210 (2.4%) seen between July and September 2017 in Victoria (Figure 1). However, M2 D31 viruses remain adamantane-resistant [9]. Original specimens were available for eight of the M2 S31 or D31 virus isolates, and sequencing confirmed 100% nt match between the isolates/clinical specimen pairs, with no evidence of mixed viral populations at the codon for position 31 of the M2 protein.

## Phylogenetic analysis of Australian M2 S31 influenza A(H3N2) viruses

**Figure 2:**
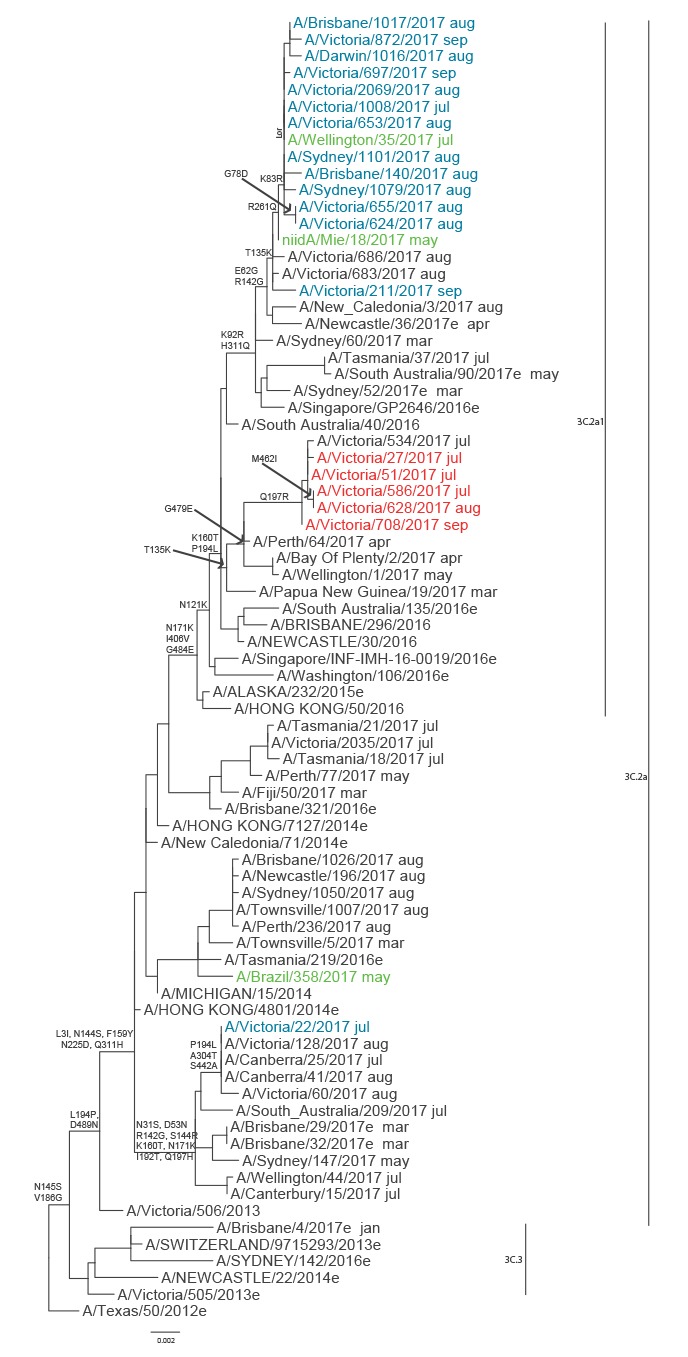
Phylogenetic tree of influenza A(H3N2) virus haemagglutinin sequences

**Figure 3:**
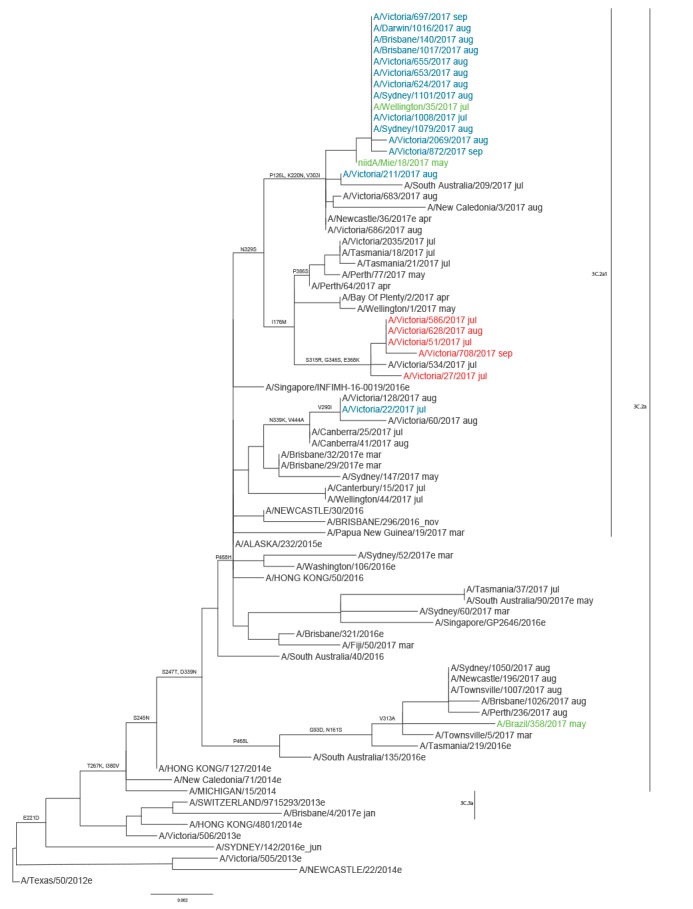
Phylogenetic tree of influenza A(H3N2) virus neuraminidase sequences

**Figure 4:**
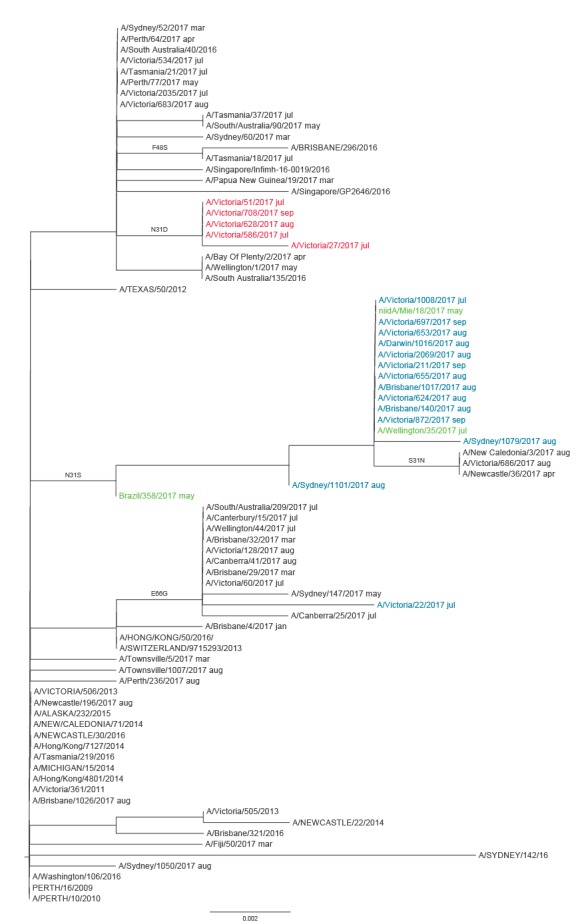
Phylogenetic tree of influenza A(H3N2) virus M2 sequences

To understand whether the M2 S31 or D31 viruses emerged from a single source or occurred sporadically, phylogenetic trees were constructed using the haemagglutinin (HA), neuraminidase (NA) and M2 gene sequences of the 2017 A(H3N2) viruses from Australia, together with global A(H3N2) virus sequences from March to September 2017 available from GISAID (Figure 2, Figure 3 and Figure 4). The authors acknowledge the originating and submitting laboratories that provided sequences to GISAID’s EpiFlu database which were used to construct the phylogenetic trees (www.gisaid.org). In both HA and NA phylogenetic trees all but two of the Australian M2 S31 viruses formed a monophyletic group, together with a Japanese and New Zealand M2 S31 virus. The Japanese virus, A/Mie/18/2017 (isolated in May 2017), was positioned ancestrally to the M2 S31 Australian viruses in both the HA and NA clades (Figure 2 and Figure 3), suggesting it may have been the progenitor. All of the M2 S31 viruses were part of the HA clade 3C.2a1, except for one strain (A/Victoria/22/2017) from July which was a 3C.2a virus (Figure 2), and all but one (A/Victoria/211/2017) of the 3C.2a1 viruses formed the subclade characterised by HA amino acid substitutions R261Q and K83R (Figure 2), which exclusively contained these viruses even when all A(H3N2) viruses via GISAID were included in the analysis. The M2 D31 viruses clustered together phylogenetically, forming a separate branch within the HA clade 3C.2a1, although this clade also contained some M2 N31 Australian viruses (Figure 2).

Australian M2 S31 viruses are coloured in blue, non-Australian M2 S31 viruses are coloured in green, and Australian M2 D31 viruses are coloured in red. All other viruses contain the N31 in the M2 protein. GISAID ID numbers for the M2 S31 and D31 virus sequences are listed in Table 2.

The sequence data were aligned using the MAFT algorithm in Geneious V10.0.09 (Biomatters Ltd, Auckland, New Zealand). Maximum likelihood phylogenetic trees were generated with RAxML v8.2, using nucleotide substitution models. Amino acid substitutions were calculated using BASEML within PAML 4.9e with FigTree v1.4.3 used to project amino acid substitutions onto the phylogenetic trees.

Australian M2 S31 viruses are coloured in blue, non-Australian M2 S31 viruses are coloured in green, and Australian M2 D31 viruses are coloured in red. All other viruses contain the N31 in the M2 protein. GISAID ID numbers for the M2 S31 and D31 virus sequences are listed in Table 2. The sequence data were aligned using the MAFT algorithm in Geneious V10.0.09 (Biomatters Ltd, Auckland, New Zealand). Maximum likelihood phylogenetic trees were generated with  RAxML v8.2, using nucleotide substitution models. Amino acid substitutions were calculated using BASEML within PAML 4.9e with FigTree v1.4.3 used to project amino acid substitutions onto the phylogenetic trees.

Australian M2 S31 viruses are coloured in blue, non-Australian M2 S31 viruses are coloured in green, and Australian M2 D31 viruses are coloured in red. All other viruses contain the N31 in the M2 protein. GISAID ID numbers for the M2 S31 and D31 virus sequences are listed in Table 2. The sequence data were aligned using the MAFT algorithm in Geneious V10.0.09 (Biomatters Ltd, Auckland, New Zealand).  Maximum likelihood phylogenetic trees were generated with RAxML v8.2, using nucleotide substitution models. Amino acid substitutions were calculated using BASEML within PAML 4.9e with FigTree v1.4.3 used to project amino acid substitutions onto the phylogenetic trees.

## Discussion and conclusion

Adamantane-resistance was first detected in persons infected with influenza virus who were treated with adamantanes in the late 1980s, in closed settings, such as nursing homes [10,11], as well as community settings [12]. In the latter, there was apparent transmission of drug-resistant strains, when rimantadine was used for treatment or post-exposure prophylaxis in families [12]. Adamantane-resistant viruses were also detected in nursing home patients without exposure to these drugs [13], demonstrating that adamantane-resistant variants may be able to spread in the community. Up to 45% of children treated with rimantadine have been reported to shed resistant viruses [14]. In addition to widespread adamantane-resistance among A(H3N2) influenza viruses circulating globally, seasonal influenza A(H1N1) viruses also developed adamantane-resistance between 2005 and 2008 [15,16], and the A(H1N1)pdm09 virus that emerged and caused the influenza pandemic in 2009 was also adamantane-resistant. As consequence of these developments, adamantanes are no longer recommended for treatment of influenza [5].

The spread of M2 N31 viruses in the early 2000s was thought to be due to linkage to advantageous substitutions elsewhere in the virus, in a process referred to as genetic ‘hitch-hiking’, and not related to adamantane-induced selection pressure [17]. Even though the A(H3N2) virus has continued to undergo substantial antigenic and genetic evolution over the last decade, the M2 N31 residue has remained almost completely fixed, suggesting that during that time it contributed to viral fitness. However, the recent detection of M2 S31 and D31 viruses in Australia suggests that the importance of the M2 N31 residue in viral fitness may no longer be as strong as it was. We encourage surveillance laboratories, where possible, to conduct M2 sequencing of A(H3N2) viruses during the upcoming 2017/18 northern hemisphere influenza season to see if the M2 S31 or D31 viruses begin to circulate in greater numbers globally.

In the seminal publication by Bright et al. on the emergence of the S31N variant in the early 2000s [4], the authors stated that ‘*further genetic and antigenic evolution of influenza A(H3N2) viruses resulting in the disappearance of the S31N mutation and reversion back to the drug sensitive phenotype should not be excluded’*. It may be that the M2 S31 viruses detected in Australasia in 2017 could be the progenitors for a reversion back to more widely circulating adamantane-sensitive A(H3N2) viruses, some 12 years after the resistant strain emerged and then dominated globally. If this were the case, it would revive the option of using adamantanes to treat A(H3N2) virus infections and improve the opportunities for using these drugs in combination with other antivirals [18].
